# Management of older adults consulting in GP surgery practices with back pain in UK Clinical Practice Research Datalink Aurum: population based study

**DOI:** 10.1186/s12891-026-09639-7

**Published:** 2026-03-02

**Authors:** Aaron Jun Yi Yap, Jessica Harris, Emma M. Clark

**Affiliations:** 1 MRC Integrative Epidemiology Unit, Population Health Sciences, Bristol Medical School, University of Bristol, Bristol, BS1 5DS UK; 2 Bristol Trials Centre, Population Health Sciences, Bristol Medical School, University of Bristol, Bristol, BS8 1NU UK; 3 Musculoskeletal Research Unit, Translational Health Sciences, Bristol Medical School, University of Bristol, Bristol, BS10 5NB UK

**Keywords:** Back pain, Radiology, Physiotherapy, Pain medication, Referral, General practice, Primary health care

## Abstract

**Background:**

The management of older adults with back pain in GP surgery practices is currently not well understood. We aimed to describe this and investigate if there are factors associated with variability in treatment.

**Methods:**

Using primary care records from UK Clinical Practice Research Datalink Aurum, we observed 594,559 adults aged 50 years and older with an index consultation for back pain between 1 January 2015 to 31 July 2018 over 18-months follow-up. Main outcome measures were time to first referral to (i) radiology, (ii) physiotherapy, (iii) exercise, physical activity programmes and musculoskeletal clinics and services, (iv) other clinics and services for further assessment, and (v) pain medication prescriptions.

**Results:**

The majority of patients received pain medication prescriptions following a back pain diagnosis (*n* = 381,829; 64.2%), but not referrals to radiology (*n* = 23,712; 4.0%), physiotherapy (*n* = 2,856; 0.5%), exercise, physical activity programmes and musculoskeletal clinics and services (*n* = 22,182; 3.7%) or other clinics and services for further assessment (*n* = 20,755; 3.5%). The probability of referrals or prescriptions at index consultation were (i) 2.6%, (ii) 0.3%, (iii) 1.7%, (iv) 1.3% and (v) 56.4% respectively. Opioids in combination with paracetamol or ibuprofen were most commonly prescribed (*n* = 151,390, 25.5%), followed by non-topical non-steroidal anti-inflammatory drugs (*n* = 127,164, 21.4%) and non-combination opioids dosage forms (*n* = 101,713, 17.1%). We observed large variability in the management of back pain within practice regions, age groups and socioeconomic status for all outcomes with the exception of pain medication prescriptions which showed little variation within factors of interest.

**Conclusions:**

Older patients presenting to GP surgery practices with back pain are typically prescribed pain medications, with few referred for imaging, further therapy or assessment. Among non-pharmacological treatments, there exist differences by practice region, age, and socioeconomic status. Future work is needed to explore reasons for these differences, and to develop new clinical guidelines or tools to facilitate the standardisation of managing back pain in older adults within GP surgery practices in the UK.

**Supplementary Information:**

The online version contains supplementary material available at 10.1186/s12891-026-09639-7.

## Background

Back pain affects more than half a billion people globally and is the leading cause of years lived with disability [1]. Back pain is known to be more common in older adults and in females, and is associated with manual occupations, current or former smoking and high body mass index (BMI), although causal mechanisms are not clearly understood [2–7]. Episodes of back pain can be recurrent, which presents challenges in care [8]. Identifying the underlying causes of back pain can be important to allow healthcare professionals to offer individualised and personalised interventions to improve health-related quality of life. While more serious underlying pathologies can occur in older adults such as vertebral fragility fractures, degenerative scoliosis and spondylolisthesis [9–11], the majority of low back pain is non-specific [12]. In the last two decades, a ‘biopsychosocial’ model has been proposed for chronic low back pain [13–16], implying that a combination of factors such as psychological stress, anxiety, depression, social network in addition to biological issues can contribute to back pain. As such, multiple studies have examined and proposed that exercise and other biopsychosocial interventions should be recommended in patients with back pain [17, 18]. In the UK, back pain was estimated to have direct costs of £1.6 billion per year and indirect costs of £10.7 billion per year in 1998, although recent estimates of direct costs have doubled [19, 20]. Despite high healthcare costs and expected increases in both prevalence and incidence of back pain with ageing populations, the management of back pain is currently not well understood or described [21, 22].

In the UK, current NICE guidelines for people aged 16 and above with low back pain do not recommend routine radiology or pharmacological therapy with the following agents: paracetamol alone, antidepressants, antiepileptics, gabapentinoids or opioids (with the exception of instances when non-steroidal anti-inflammatory drugs (NSAIDs) are not appropriate for use in treatment of acute episodes) [23]. A risk-stratified approach is recommended, such as self-management in mild cases, and exercise programmes with or without oral NSAIDs in more severe or complex cases. However, it is known that clinicians do not always adhere to these guidelines, citing guideline rigidity as one of the primary reasons for deviation [24, 25]. In addition, these guidelines are only for low back pain and not pain in other areas of the spine.

Hence, we aimed to describe the ‘usual care’ of older adults consulting in GP surgery practices with back pain using the Clinical Practice Research Datalink (CPRD) Aurum database. Our objectives were to: (1) describe the characteristics of older patients presenting to GP surgery practices with back pain and their healthcare pathways, (2) report the time to the first referrals to (i) radiology, (ii) physiotherapy, (iii) exercise, physical activity programmes and musculoskeletal (EPaMsk) clinics and services, (iv) other clinics and services for further assessment (at secondary care), and (v) pain medication prescriptions following an index consultation for back pain, and 3) investigate if there are factors associated with variations in treatment patterns. The motivation of this work was to fill the current knowledge gap – to understand treatment patterns of older patients who visit GP surgery for an episode of back pain. In addition, we had envisioned the study to describe the ‘usual care’ which is important when considering and planning for the control arm of any future Randomised Controlled Trial (RCT) for management of back pain [26].

## Methods

### Study design, setting and participants

The study design was a population-based cohort study including all adults aged 50 years and above with an index consultation at GP surgery practice for back pain between 1 January 2015 to 31 July 2018. We decided to set the threshold of 50 years for an ‘older adult’ because guideline recommendations for osteoporosis screening commonly begin at age 50 [27]. Back pain was defined as the presence of a diagnosis code suggesting lumbar, thoracic or a general non-specific code of back pain; this was done to maximise the generalisability and inclusion of patients given that there were many observations of general non-specific codes of back pain in the GP surgery setting. Clinicians can type and select from a list of codes in the information system; the list of codes used to define back pain in the study are available in Supplementary Table 1. Patients were required to have at least 1-day of records of any nature in the database from the start of follow-up (date of index consultation for back pain). This was done to ensure that each patient could contribute to observable person-time or time at risk. We also only included patients with an ‘acceptable’ flag within the CPRD Aurum database; this is a data quality process conducted by CPRD to ensure that patients’ records are of an appropriate quality for research (e.g., having sensible values and non-missing data for registration dates, date of birth, age and gender) [28].

As we wanted to ensure that the initial back pain visit was an incident episode, we applied a 1-year washout period: patients with coded visits of back pain 1-year before the date of the identified back pain episode were excluded. Patients were followed from the date of index consultation until the earliest of the following: end of 18-months follow-up, death, practice transfer or when they had the outcomes of interest.

As we were certain that COVID-19 pandemic measures would impact treatment patterns and patients’ presentation to GP surgeries for back pain, we decided to limit follow-up to within ‘pre-COVID’ periods; hence the last date of entry to the cohort was 31 July 2018 to allow for an 18-months follow-up until 31 January 2020 [29].

### Data source

Our data source was CPRD Aurum, which is a primary care record database comprising of GP surgery practices using ‘EMIS Health’ systems in the UK, with more reliable records available from 1995 [30]. ‘EMIS’ (formerly known as Egton Medical Information Systems) was an electronic health record provider which was acquired by ‘Optum’ in 2023 which currently still maintains ‘EMIS’ in the name of their products as at the time of writing [31]. CPRD Aurum has information on demographics, diagnoses, symptoms, prescriptions, referrals, immunizations, tests and results [32]. We used the July 2023 build, which covered approximately 16% of the UK population (10,780,368 current patients) and 14% of UK general practices (1,164 currently contributing practices), with no data from practices in Scotland or Wales [33].

CPRD Aurum also has an existing algorithm for ascertaining death dates [34]. In this study, we defined death dates as the earlier of the following: date of death as recorded in EMIS Health by the GP surgery practice or as ascertained by the CPRD algorithm.

### Covariates

We pre-determined a list of covariates which we thought could potentially influence the management of back pain: age, gender, ethnicity, BMI, socioeconomic status (SES), smoking status, alcohol use, comorbidity burden, osteoporosis, vertebral fracture and practice region. These were chosen in consultation with a practicing clinician (EMC) and based on previously known risk factors reported in literature [2, 4, 5, 12, 16]. These covariates were ascertained at baseline (date of index consultation for back pain). A summary of the list of covariates is available in the data supplement (Supplementary Table 2).

Age at index consultation was categorized into the following categories: 50–59 years, 60–69 years, 70–79 years and 80 years and older. Ethnicity was determined by coded records in the CPRD observation tables and was grouped into five categories: White, South Asian, Black, Other, Mixed. We adopted an algorithm from an online repository [35]: the most frequently coded ethnicity category was kept, followed by the most recently coded category if there were two or more categories with the same counts, otherwise we treated the data as missing. BMI was adapted from a published algorithm [36]; we calculated BMI from the most recently recorded height and weight values before or on the index consultation date, followed by coded BMI values if height and weight values were not available. BMI values outside the range of 9–180 kg/m^2^ were dropped. Further details for all covariates are available in the data supplement (Supplementary Text 1). BMI was classified into five categories with reference to suggested ranges for White, Hispanic and Black individuals by the National Institute of Health and World Health Organization [37, 38]. We used practice level index of multiple deprivation (IMD) as a proxy for socioeconomic status as individual level IMD was not available at the point of conducting the study. SES was classified into five quintiles, with Quintile 1 being the most deprived. For smoking status and alcohol use, we retained the nearest coded record at any time before (from 1995) or equal to the index consultation date. If there was a previous record of smoking, we re-categorised non-smokers as ex-smokers. If there were multiple categories on the same date, we took the next most recent record of smoking status, otherwise we considered the data to be missing. Code lists for both smoking status and alcohol use were adapted from an online repository [35]. In initial exploratory analyses we observed that alcohol use had a high proportion of missing data, hence we decided to exclude alcohol use from any regression models although we have reported this in descriptive analyses.

Comorbidity burden, osteoporosis and vertebral fracture were defined by examining observational records up to 1-year before the index consultation date. Comorbidity burden was measured by the Charlson comorbidity index (CCI) [39]. For CCI, we used code lists which were internally validated in a previous study. We grouped comorbidity burden into three categories: None (CCI score of 0), Low (CCI score of 1–2) and High (CCI score of 3 or more). Osteoporosis and vertebral fracture codes were reviewed by a practicing clinician for accuracy (EMC).

### Outcomes

We were interested to examine management of back pain at first presentation to GP surgery: referrals to (i) radiology, (ii) physiotherapy, (iii) EPaMsk clinics and services, (iv) other clinics and services for further assessment, and (v) pain medication prescriptions. Physiotherapy codes were indicative of formal supervision by a physiotherapist, whereas ‘exercise and physical activity programmes’ codes were suggestive of self-directed management. In initial specification of the protocol, we had decided to group ‘exercise and physical activity programmes’ with ‘musculoskeletal clinics and services’ as the former had low number of events in initial exploratory analyses and we had conceptualised these as ‘intervention-based’ approaches. However, we recognised that this grouping was not ideal upon further clinical consultation as the latter is likely to broadly contain interface services between primary and secondary care. Hence, we decided to conduct a post-hoc analysis separating ‘exercise and physical activity programmes’ with ‘musculoskeletal clinics and services’. (See section *‘Post-hoc analysis’*). ‘Other clinics and services for further assessment’ consisted of referrals to secondary care including back pain clinics, falls service or assessment, fracture clinic, general medicine clinic, geriatric or older people services, orthopaedics, osteoporosis clinics, pain clinics, rheumatology and spinal teams.

We defined referrals as the presence of observations found in the ‘Referral’ table of CPRD Aurum or if codes suggested the referral action (e.g., refer to Radiology). This approach was informed by a previous study which supported the idea of adding codes with a description of a referral to Aurum referral records to improve consistency and comprehensiveness [40]. Referrals in CPRD Aurum are coded by the clinician and involves external care centres including interface services between primary and secondary care, and secondary care locations such as hospitals for outpatient or inpatient care [41]. To ensure that referrals were relevant to the back pain episode, we included only records which were made during the same consultation visit with a diagnosis of back pain; a same consultation visit was defined as having the same consultation identification (ID) or if the referral was coded on the same day or one day apart from the date of consultation for back pain. We decided on a one day allowance as during exploratory analyses we observed that there were referral records with the same consultation ID as the back pain episode but were coded a day apart (both a day before or after).

For pain medication prescriptions, we included all medications containing paracetamol, NSAIDs, opioids, gabapentinoids, antidepressants commonly used for pain, benzodiazepines and non-benzodiazepine hypnotics. Antidepressants included amitriptyline, citalopram, duloxetine, fluoxetine, paroxetine and sertraline which were recommended by NICE for management of chronic pain, and nortriptyline which is commonly used for pain [42]. As we recognised that these pain medications could be prescribed for reasons other than for back pain, we included prescriptions only if they were made during the same consultation visit: if the prescription was made on the same day or 1-day apart from the date of consultation for back pain. In statistical models, we included all recorded prescriptions. We also reported the proportion of prescriptions which were incident for each medication class. Incident use was defined as patients having no recorded prescription within 1-year from index consultation date with respect to drug substance.

Code lists for outcomes were constructed by wildcard search of terms and drug substance names in the CPRD Aurum code browser. These were also reviewed by a practician clinician for accuracy (EMC). Inclusion and exclusion criteria for pain medication prescriptions are detailed in the data supplement (Supplementary Table 4).

### Statistical analyses

We pre-published a statistical analysis plan during planning phases of the study which is available online [43]. All analyses were conducted in R version 4.4.1. Characteristics of patients were reported at baseline which was the date of index consultation for back pain. We had planned to include only complete cases in our analyses; to understand if there were differences between complete cases and those with missing data, we reported the baseline characteristics in both stratums and computed standardised mean differences (SMD) using R package ‘*tableone*’ [44]. Data were defined to be missing if there were unknown or incomplete information on gender, ethnicity, BMI or smoking status variables.

To map the healthcare pathways of patients presenting with back pain, we grouped referrals to physiotherapy, EPaMsk clinics and services and other clinics and services for further assessment together due to relatively small number of patients who experienced the outcomes.

We administratively censored patients when they died, transferred practice, had the outcomes of interest or reached the end of the 18-month follow-up period. Analyses used the R ‘*survival*’ package [45]. For each outcome, we reported the total number of events, total follow-up time and the unadjusted probability of experiencing the outcome at 0-days, 180-days and 365-days following a Kaplan-Meier approach. For pain medication prescriptions, we reported this for all medications; analyses by medication class are available in the data supplement. We further reported the proportion of prescriptions which were incident and the total number of patients which had incident prescriptions; this was calculated by dividing the number of incident prescriptions and patients (with incident prescriptions) over the total number of prescriptions and patients respectively.

To investigate factors which were associated with variations in treatment, we added all covariates which we had thought to potentially influence the management of back pain into Cox regression models; we did not assess for collinearity or interaction. We reported the corresponding hazard ratios with 95% confidence intervals (CI).

### Post-hoc analysis

We conducted a single post-hoc analysis stratifying EPaMsk into two distinct categories: ‘exercise and physical activity programmes’ and ‘musculoskeletal clinics and services’. This was conducted as a sensitivity analysis to examine for differences between management modalities given that ‘musculoskeletal clinics and services’ could contain interface services between primary and secondary care. Results were reported in Supplementary Tables 12–13.

### Ethical approval

The study protocol was approved via the Clinical Practice Research Datalink Research Data Governance process (protocol number: 20_000235).

## Results

### Study population characteristics

**Fig. 1:**
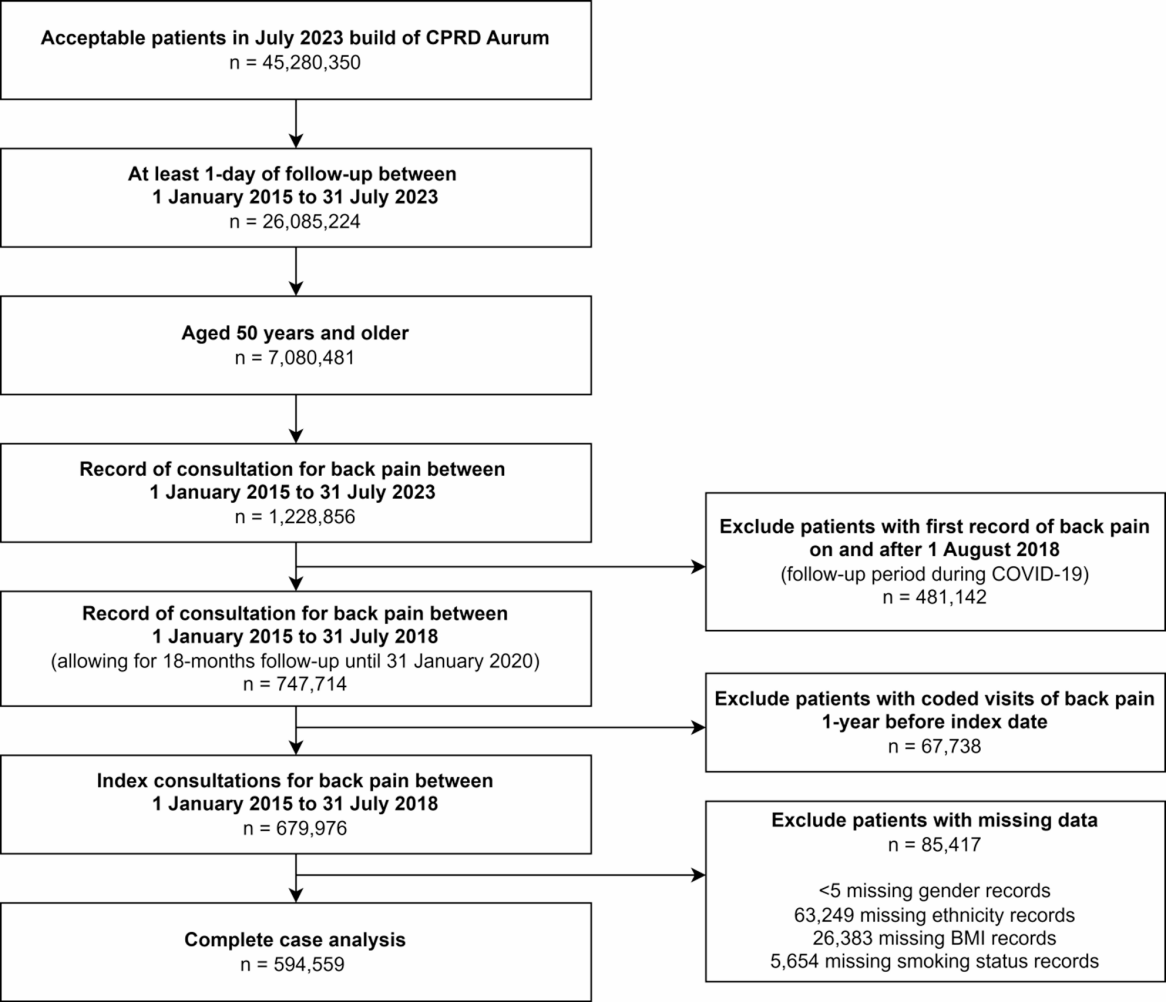
Flow chart of cohort inclusion and exclusion. BMI: Body mass index

From 1 January 2015 to 31 July 2018, we identified 679,976 patients aged 50 years and above who presented to their GP surgery for an index episode of back pain, of which 594,559 (87.4%) were complete cases (Fig. 1). Table 1 shows the characteristics of these patients. In the complete case cohort, 57.1% were female and 88.5% were of white ethnicity. The median age was 65 years. The majority of patients (91.7%) were observed for the full 18-month follow-up period, with 3.4% of loss to follow-up attributed to deaths. During follow-up, approximately a-third of patients (38.1%) returned to the GP for at least one repeat consultation for back pain.

**Table 1:** Characteristics of patients with an index consultation for back pain from January 2015 to July 2018 in CPRD Aurum

Characteristics	Study population (*n* = 679976)	Complete case cohort (*n* = 594559)	Missing data cohort† (*n* = 85417)	Standardised mean differences
Outcomes				
Referred to radiology (%)	26,997 (4.0)	23,712 (4.0)	3285 (3.8)	0.01
Referred to physiotherapy (%)	3316 (0.5)	2856 (0.5)	460 (0.5)	0.01
Referred to exercise or physical activity programmes, musculoskeletal clinics and services (%)	24,563 (3.6)	22,182 (3.7)	2381 (2.8)	0.05
Referred to other clinics and services for further assessment* (%)	23,401 (3.4)	20,755 (3.5)	2646 (3.1)	0.02
Prescriptions for pain (%)	436,523 (64.2)	381,829 (64.2)	54,694 (64.0)	< 0.01
Exposures and covariates at baseline				
Age at index consultation, years				
Mean (SD)	66.6 (11.2)	66.3 (10.9)	68.4 (12.9)	0.17
Median (IQR)	65 (57–75)	65 (57–74)	66 (57–79)	
Range	50–110	50–106	50–110	
Female† (%)	385,791 (56.7)	339,306 (57.1)	46,485 (54.4)	0.05
Ethnicity † (%)				
White	546,683 (80.4)	526,435 (88.5)	20,248 (91.3)	0.13
South Asian	36,097 (5.3)	35,316 (5.9)	781 (3.5)	
Black	22,531 (3.3)	21,923 (3.7)	608 (2.7)	
Other	6901 (1.0)	6543 (1.1)	358 (1.6)	
Mixed	4515 (0.7)	4342 (0.7)	173 (0.8)	
Practice region (%)				
North East	28,949 (4.3)	27,140 (4.6)	1809 (2.1)	0.30
North West	145,115 (21.3)	128,403 (21.6)	16,712 (19.6)	
Yorkshire And The Humber	24,712 (3.6)	21,236 (3.6)	3476 (4.1)	
East Midlands	16,549 (2.4)	14,657 (2.5)	1892 (2.2)	
West Midlands	116,274 (17.1)	103,602 (17.4)	12,672 (14.8)	
East of England	30,716 (4.5)	26,318 (4.4)	4398 (5.1)	
London	100,772 (14.8)	91,951 (15.5)	8821 (10.3)	
South East	140,651 (20.7)	117,569 (19.8)	23,082 (27.0)	
South West	74,272 (10.9)	62,577 (10.5)	11,695 (13.7)	
Wales	0	0	0	
Scotland	0	0	0	
Northern Ireland	1966 (0.3)	1106 (0.2)	860 (1.0)	
Socioeconomic status (%)				
Quintile 1 (Lowest)	118,324 (17.4)	100,981 (17.0)	17,343 (20.3)	0.17
Quintile 2	112,639 (16.6)	96,920 (16.3)	15,719 (18.4)	
Quintile 3	136,068 (20.0)	117,672 (19.8)	18,396 (21.5)	
Quintile 4	155,735 (22.9)	136,794 (23.0)	18,941 (22.2)	
Quintile 5 (Highest)	157,210 (23.1)	142,192 (23.9)	15,018 (17.6)	
Body mass index†, kg/m^2^				
Mean (SD)	28.4 (5.9)	28.5 (5.9)	27.6 (5.9)	0.15
Median (IQR)	27.6 (24.4–31.5)	27.7 (24.5–31.6)	26.8 (23.7–30.6)	
Range	9.1-178.6	9.1–160.0	9.1-178.6	
Comorbidity burden (%)				
High (CCI score ≥ 3)	19,767 (2.9)	16,994 (2.9)	2773 (3.2)	0.10
Low (CCI score 1–2)	182,327 (26.8)	162,479 (27.3)	19,848 (23.2)	
None (CCI score 0)	477,882 (70.3)	415,086 (69.8)	62,796 (73.5)	
Osteoporosis (%)	11,362 (1.7)	9673 (1.6)	1689 (2.0)	0.03
Vertebral fracture (%)	529 (0.1)	436 (0.1)	93 (0.1)	0.01
Smoking status† (%)				
Active smoker	105,272 (15.5)	91,435 (15.4)	13,837 (17.3)	0.07
Ex-smoker	216,120 (31.8)	192,241 (32.3)	23,879 (29.9)	
Non-smoker	352,930 (51.9)	310,883 (52.3)	42,047 (52.7)	
Alcohol use (%)				
High	9601 (1.4)	7921 (1.5)	1680 (3.0)	0.18
Moderate	68,231 (10.0)	61,603 (12.0)	6628 (11.7)	
Low	437,069 (64.3)	396,637 (77.4)	40,432 (71.6)	
Non-drinker	54,221 (8.0)	46,491 (9.1)	7730 (13.7)	
Observed for full 18-months follow-up (%)	614,231 (90.3)	545,063 (91.7)	69,168 (81.0)	0.32
Died during 18-months follow-up (%)	27,700 (4.1)	20,051 (3.4)	7649 (9.0)	0.23
Registration at practice ended before end of follow-up (%)	38,550 (5.7)	29,463 (5.0)	9087 (10.6)	0.21
Data collection by CPRD ended before end of follow-up (%)	3163 (0.5)	2596 (0.4)	567 (0.7)	0.03
Follow-up duration, months				
Mean (SD)	17.1 (3.2)	17.2 (3.0)	16.2 (4.5)	0.28
Median (IQR)	18 (18–18)	18 (18–18)	18 (18–18)	
Range	0–18	0–18	0–18	
Back pain consultations during follow-up, *n*				
Mean (SD)	1.8 (1.6)	1.8 (1.6)	1.8 (1.6)	0.02
Median (IQR)	1 (1–2)	1 (1–2)	1 (1–2)	
Range	1–51	1–51	1–30	
Back pain consultations during follow-up, *n* (%)				
1 coded visit	422,492 (62.1)	368,229 (61.9)	54,263 (63.5)	0.03
2 coded visits	137,474 (20.2)	120,708 (20.3)	16,766 (19.6)	
3 coded visits	56,715 (8.3)	49,995 (8.4)	6720 (7.9)	
≥ 4 coded visits	63,295 (9.3)	55,627 (9.4)	7668 (9.0)	

Complete cases were similar to patients with missing data especially for our outcomes of interest, although there were differences observed by practice region (SMD = 0.30), duration of follow-up (SMD = 0.28) and death (SMD = 0.23)

*CCI* Charlson comorbidity index, *IQR *interquartile range, *SD *standard deviation

* Includes referrals to back pain clinic, falls service or assessment, fracture clinic, general medicine clinic, geriatric or older people services, orthopaedics, osteoporosis clinics, pain clinics, rheumatology or spinal team

† Contains missing data (Gender: *n* < 5, Ethnicity: *n* = 63249 (9.3%), BMI: *n* = 26383 (3.9%), Smoking status: *n* = 5654 (0.8%)). Complete cases were defined as not having missing data for gender, ethnicity, BMI and smoking status

### Healthcare pathway

**Fig. 2:**
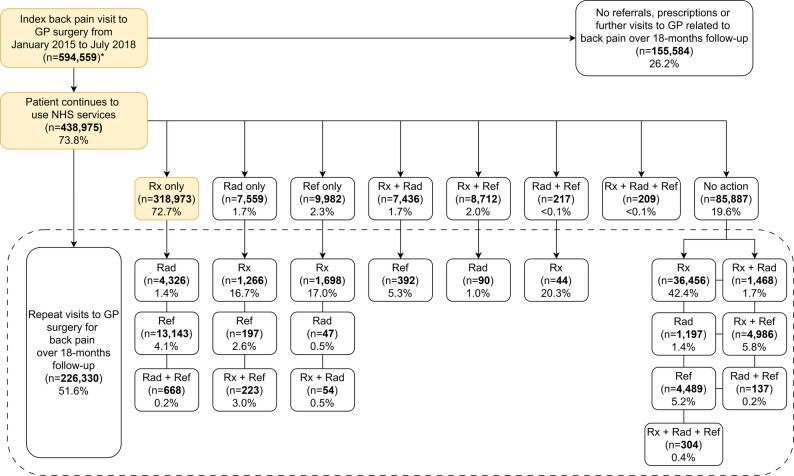
Healthcare pathway from index consultation of back pain in GP surgery practices in CPRD Aurum (2015–2018). Rx: pain medication prescription; Rad: referral to radiology; Ref: referral to physiotherapy, exercise or physical activity programmes, musculoskeletal clinics and services, back pain clinics, falls services or assessments, fracture clinics, general medicine clinics, geriatric or older people services, orthopaedics, osteoporosis clinics, pain clinics, rheumatology or spinal teams. *Complete cases only: no missing data. This figure shows the sequence of various treatments for back pain following the index consultation in GP surgery practices in CPRD Aurum. The most common healthcare pathway for management of back pain is shaded in yellow. Percentages shown are calculated by taking the number of individuals in the group of interest divided by the number of individuals in the group a level above

Figure 2 illustrates the different treatment pathways for patients in our cohort. Pain medication prescriptions alone during the index consultation was the most common pathway (*n* = 318,973, 53.6%), with only 18,137 (5.7%) patients from this subset receiving referrals to radiology, physiotherapy, EPaMsk clinics and services, and other clinics and services for further assessment during repeated visits for back pain. Approximately 40% (*n* = 241,471) of patients had no prescriptions or referrals at the index consultation; a-third of these patients (*n* = 85,887) subsequently returned to their GP for repeated visits, with 57% (*n* = 49,037) receiving prescriptions/referrals during these repeated visits.

Among all patients who returned to the GP for at least one repeat consultation, 31.5% (*n* = 71,185) had a new management modality, of which 44.6% (*n* = 31,721) were attributed to non-pharmacological management.

### Time to first referrals and pain medication prescriptions

Approximately two-thirds of the cohort were prescribed pain medications during follow-up (*n* = 381,829, 64.2%) (Table 2). In contrast, it was not routine practice (≤ 4%) to be referred to radiology, physiotherapy, EPaMsk clinics and services, or other clinics and services for further assessment. The probability of referrals to (i) radiology, (ii) physiotherapy, (iii) EPaMsk clinics and services, (iv) other clinics and services for further assessment and (v) pain medication prescriptions at the index consultation were 2.6%, 0.3%, 1.7%, 1.3% and 56.4% respectively; this increased marginally at one-year from the date of index consultation: 3.8%, 0.5%, 3.4%, 3.2% and 63.2% respectively. Stratified results by age group, gender, SES and comorbidity burden, and for time to first pain medication prescription by medication class are available in the data supplement (Supplementary Tables 5–10). We observed some relative differences across strata, but absolute differences were small.

**Table 2:** Time to the first referral to outcomes during 18-months follow-up*

Outcome	Patients with referrals or prescriptions, *N* (%)	Follow-up time (100 py)	Pr of referral or prescription at 0-days, % (95% CI)	Pr of referral or prescription at 180-days, % (95% CI)	Pr of referral or prescription at 365-days, % (95% CI)
Radiology	23,712 (4.0)	8218	2.6 (2.6–2.6)	3.5 (3.5–3.6)	3.8 (3.8–3.9)
Physiotherapy	2856 (0.5)	8489	0.3 (0.3–0.3)	0.4 (0.4–0.4)	0.5 (0.4–0.5)
Exercise or physical activity programmes, musculoskeletal clinics and services	22,182 (3.7)	8251	1.7 (1.6–1.7)	3.1 (3.0-3.1)	3.4 (3.4–3.5)
Other clinics and services for further assessment†	20,755 (3.5)	8272	1.3 (1.3–1.4)	2.8 (2.8–2.9)	3.2 (3.2–3.3)
Pain medication prescriptions	381,829 (64.2)	3249	56.4 (56.3–56.5)	61.5 (61.4–61.6)	63.2 (63.0-63.3)

*CI* confidence interval, *Pr *probability, *py *person-years

*N = 594,559

† Includes referrals to back pain clinic, falls service or assessment, fracture clinic, general medicine clinic, geriatric or older people services, orthopaedics, osteoporosis clinics, pain clinics, rheumatology or spinal team

### Pain medication prescriptions by medication class

**Fig. 3:**
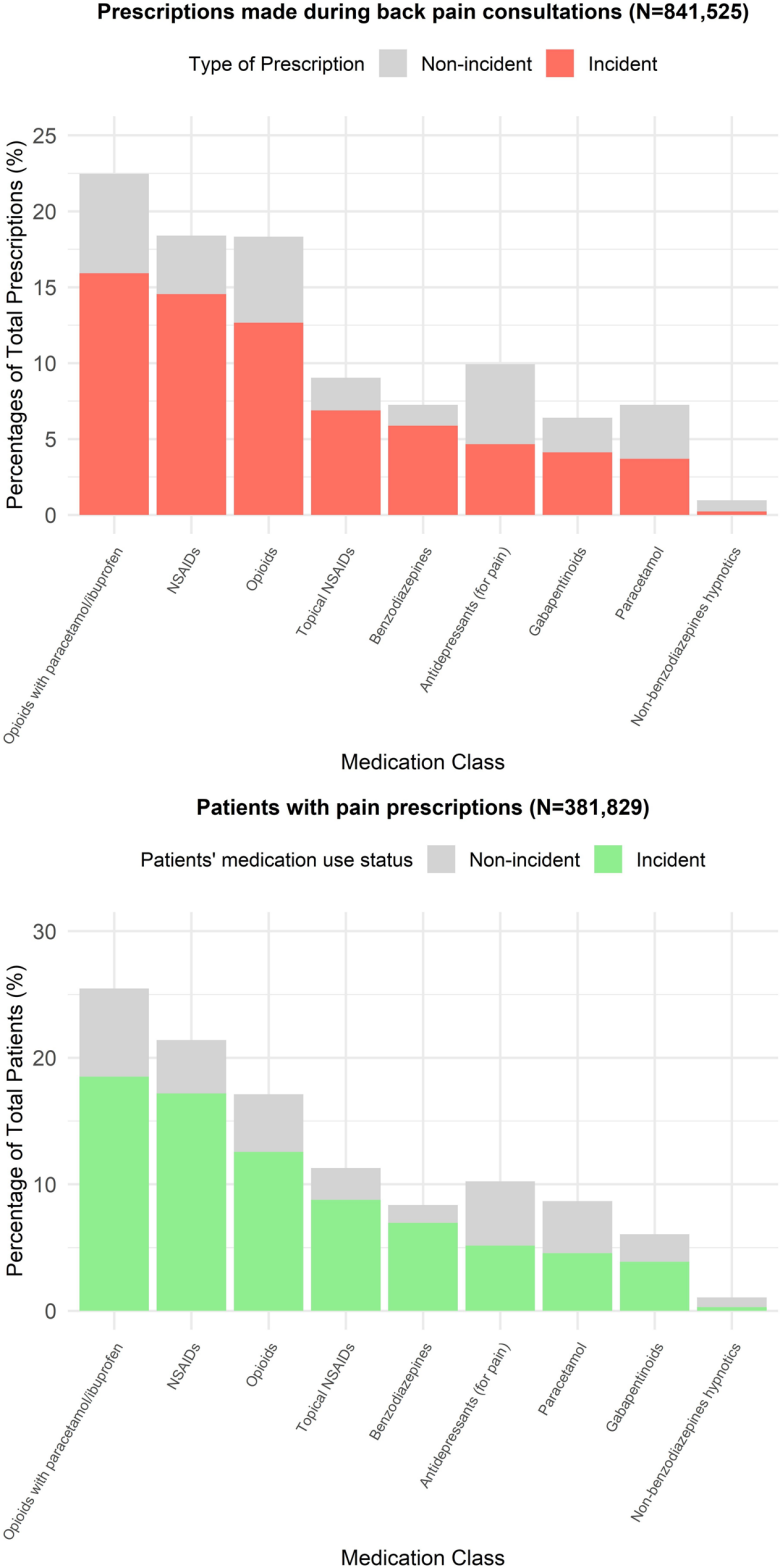
Prescriptions for back pain during 18-months follow-up, stratified by incident use. The top figure shows the proportion of prescriptions prescribed during back pain consultations, grouped by medication class. The bottom figure shows the proportion of patients prescribed medications during back pain consultations, grouped by medication class. Medication classes are ordered by number of prescriptions or patients which were incident. Incident use was defined as not having any prescription of the drug substance 1-year prior to index consultation

Opioids in combination with paracetamol or ibuprofen were most commonly prescribed, followed by non-topical NSAIDs and non-combination opioids dosage forms (Table 3; Fig. 3). Overall, 68.6% and 81.7% of prescriptions and patients respectively were associated with incident use (no prescription of drug substance 1-year prior to index consultation). Proportion of incident use was highest with benzodiazepines, followed by non-topical NSAIDs and topical NSAIDs. Non-benzodiazepine hypnotics such as Zopiclone and Zolpidem had the lowest proportion of incident use: a large proportion of patients who were prescribed non-benzodiazepine hypnotics during the index consultation for back pain were already existing users (had records of use in the year before).

**Table 3:** Pain medication prescriptions by medication class for older adults presenting with back pain

	All prescriptions		Incident use*		Proportion of incident over total	
	Prescriptions during consults, *N*	Patients, *N* (%)	Prescriptions during consults, *N*	Patients, *N* (%)	Prescriptions during consults, %	Patients, %
Total	841,525	381,829 (64.2)	576,941	312,132 (52.5)	68.6	81.7
(1) Paracetamol	60,955	51,533 (8.7)	31,062	27,041 (4.5)	51.0	52.5
(2) NSAIDs	154,812	127,164 (21.4)	122,406	102,102 (17.2)	79.1	80.3
(3) Topical NSAIDs	76,066	67,031 (11.3)	57,900	52,067 (8.8)	76.1	77.7
(4) Opioids in combination with paracetamol or ibuprofen	189,012	151,390 (25.5)	133,947	109,981 (18.5)	70.9	72.6
(5) Opioids	154,137	101,713 (17.1)	106,484	74,652 (12.6)	69.1	73.4
(6) Gabapentinoids	53,889	35,908 (6.0)	34,673	23,033 (3.9)	64.3	64.1
(7) Antidepressants (for pain)	83,568	60,786 (10.2)	39,173	30,627 (5.1)	46.9	50.4
(8) Benzodiazepines	60,963	49,655 (8.4)	49,406	41,337 (7.0)	81.0	83.2
(9) Non-benzodiazepines hypnotics	8123	6281 (1.1)	1890	1687 (0.3)	23.3	26.9

*Incident use was defined as not having any prescription of the drug substance 1-year prior to index consultation

### Variation in management of back pain

**Fig. 4:**
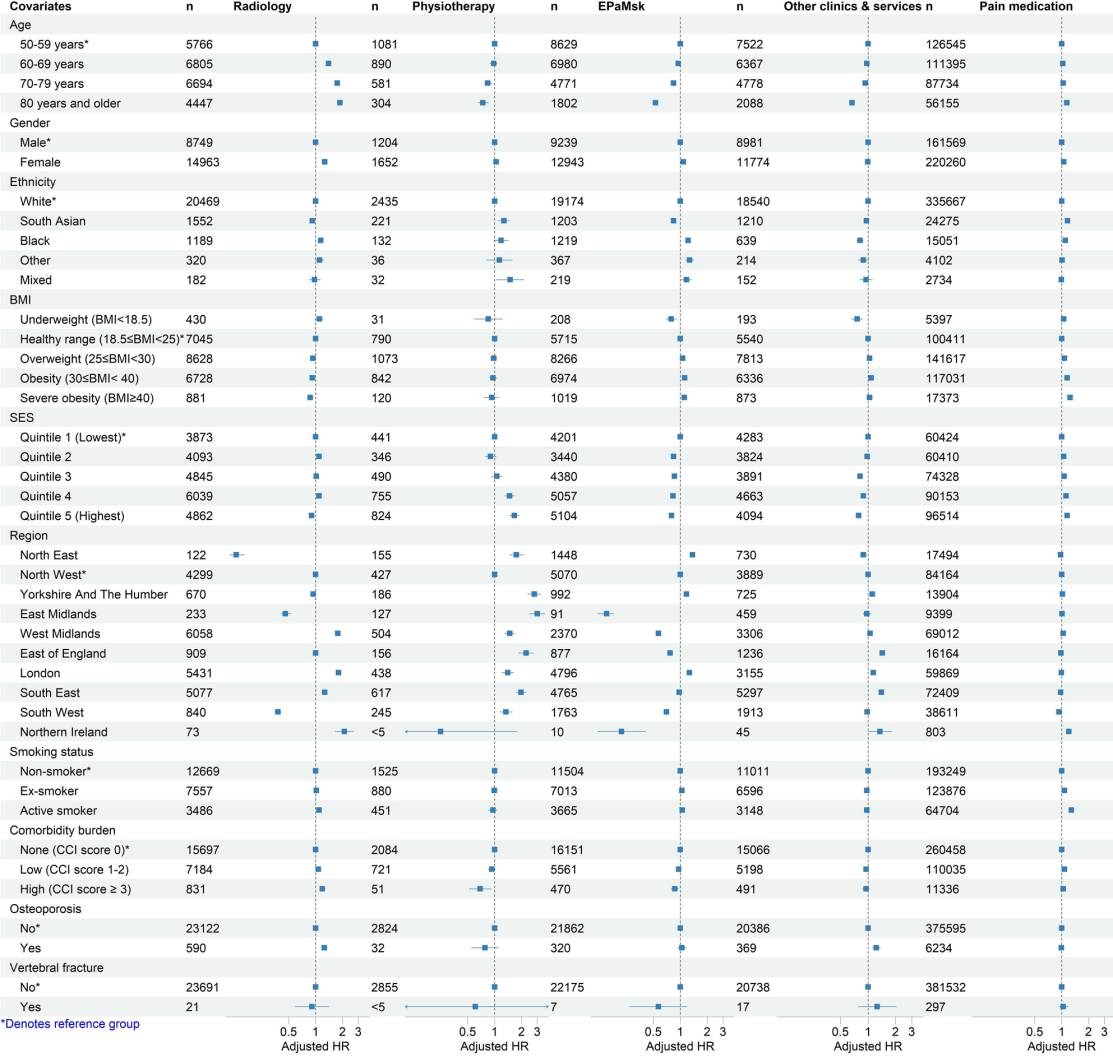
Cox regression models of covariates and time to first referrals and pain medication prescriptions. BMI: body mass index; EPaMsk: Exercise, physical activity programme and musculoskeletal; HR: hazard ratio; SES: socioeconomic index

In our Cox regression models, we observed variability in the management of back pain within covariates of interest, with the exception of pain medication prescriptions (Fig. 4). We observed the largest variability by practice region for referrals to radiology, physiotherapy, EPaMsk clinics and services, and other clinics and services for further assessment. Patients who were older or who had more comorbidities were more likely to receive a referral to radiology, but less likely to be referred to physiotherapy, EPaMsk clinics and services, and other clinics and services for further assessment. Females had a higher likelihood of being referred to radiology as compared to males. We also observed that patients who had higher SES were more likely to be referred to physiotherapy, but less likely to EPaMsk clinics and services, and other clinics and services for further assessment. A table of the detailed results and estimates are available in the data supplement (Supplementary Table 11).

### Comparing exercise and physical programmes with musculoskeletal clinics and services

In the post-hoc analysis, we observed that there were much fewer patients referred to exercise and physical activity programmes (*n* = 498, < 0.1%) as compared to musculoskeletal clinics and services (*n* = 21,736, 3.7%) (Supplementary Table 12). Patients with higher BMI were more likely to be referred to exercise and physical activity programmes, suggesting a possible focus on weight-related management.

## Discussion

In this study, we were interested to understand the ‘usual care’ of older adults consulting in GP surgery practices with back pain, and if there were factors which were associated with variability in treatment across the UK. We observed that patients are commonly just prescribed pain medications at the index consultation, with few patients referred for non-pharmacological treatment or for further investigation by radiology or by other clinics and services. This could be appropriate in less severe cases and is in line with common opinion that unnecessary referrals for back pain might be wasteful and unwarranted [23, 46–50]. However, studies have shown that challenges persist in both the identification of suitable candidates for referrals, and the accurate diagnosis of more severe conditions such as vertebral fracture and spinal malignancy [51–54]. Despite as many as more than a-third of patients in our cohort returning for repeat consultations for back pain, less than one-fifth of patients are referred to a new non-pharmacological management modality. Furthermore, we found that there exists a large variation in non-pharmacological management by practice region and SES (age group variation is expected), which suggests there exists inequalities and inconsistencies in how back pain is managed in GP surgery practices in the UK. A recent study using a different data source also observed variability of physical activity referrals by region in England patients diagnosed with non-communicable diseases [55]. This necessitates further investigation into the potential reasons for the observation of inequalities, the development of newer clinical guidelines to address these inequalities, and the implementation of suitable strategies or tools to facilitate the standardisation of practice within GP surgery practices in the UK.

In contrast, we found medications for pain to be widely prescribed in the cohort, with pharmacological therapy in general having little variability within factors examined in the study. Weak opioids in combination with paracetamol/ibuprofen and non-topical NSAIDs were most commonly prescribed, which is consistent with the general recommendations of guidelines [23]. However, we observe that a fair number of patients had received incident prescriptions of opioids, gabapentinoids and benzodiazepines during consultation for back pain, which is inconsistent with current recommendations. This is likely attributed to the time period of study and follow-up, i.e., before guidelines were updated with new recommendations and strategies to tackle dependence or withdrawal, alongside better awareness of the ‘opioid epidemic’ in the UK [56–59]; A future study describing more recent medication use could clarify this observation. Prescriptions of non-benzodiazepine hypnotics were likely associated with prevalent use rather than the index episode of back pain.

Rather than relying on a pain medication prescription, management of back pain in older adults should start with an assessment of alternative diagnoses according to NICE guidance [23]. In the UK, this can be first assessed by musculoskeletal First Contact Practitioners (FCP) who can provide recommendations on exercise and advice, and triage to determine if further referrals are required to General Practitioners, secondary care orthopaedic, rheumatology and pain clinics, and physiotherapy in community or secondary care [60]. This is in line with the ‘biopsychosocial’ model proposed which suggests that back pain is multi-factorial [13–16]. A related challenge is knowing who to refer for spinal radiographs, given that 4–14% of older adults have a vertebral fragility fracture related to osteoporosis [9], and that the majority of people with these fractures experience long term back pain [61]. Some risk stratification tools or guidance are available, and can be used to identify patients who are at high risk of developing more serious biological pathologies [62, 63].

Treatment recommendations within the NICE guidance also provide advice and information on self-management. This was not coded within CPRD; hence we were unable to assess if this was undertaken. Exercise and/or physical programmes should be recommended to patients with specific episodes or flares of low back pain or patients at higher risks of poor outcomes, with consideration of their underlying medical conditions and needs. We found this to be uncommon in our study. If pharmacological therapy is indicated, oral NSAIDs at the lowest effective dose for the shortest duration should be the choice of therapy unless patients have active cardiovascular, renal, gastrointestinal or liver issues, in which case weak opioids with or without paracetamol can be considered.

Our study had several strengths: this is the first study to our knowledge which describes the ‘usual care’ of back pain in GP surgery practices using large electronic health records data, which better reflects real-world clinical practice as compared to RCTs and allows for assessment of adherence to guidelines. Results are largely generalisable to the UK but not Wales and Scotland as we had no data from GP practices in these regions. Loss to follow-up in this study was low, with more than 91% of patients observed for the full follow-up duration of the study. In addition, we had attempted to minimise misclassification by ascertaining outcomes which were related to the back pain episode by including only records which were coded during back pain consultations, such as referrals made at the GP. However, this would have relied on diligent coding of back pain diagnoses and referrals records; If back pain diagnoses and referrals were ‘under-coded’, we would anticipate our outcome numbers to be underestimated, because we had limited our observation of outcomes to only referrals made during back pain consultations. We did however identify a large number of patients with pain medication prescriptions, which we found reassuring. Our study also did not account for actual attendances at radiology, physiotherapy or clinics and services, which implies that reported numbers could be slightly lower if we assume that not all patients attended their treatments. We also note that estimates reported in Cox regression models may not be total effects of each covariate and readers should interpret the point estimates and confidence intervals with caution; hence our focus in this study was describing the direction and relative magnitudes rather than discussing specific estimates [64]. In addition, we had not included depression and anxiety as covariates in the model as this was not part of the pre-specified analysis plan, although this could be examined in a future study. We also recognise findings of this study may not be generalisable to post-COVID-19 periods as we had only examined pre-COVID periods. Finally, we did not have data on occupations which is likely an important factor to consider when modelling variability, although we think that it is unlikely to affect the variability that we observe by practice region.

This study shows that although back pain appears to be a relatively ‘simple’ and non- life-threatening condition experienced by many older adults, there exists significant variation in the management of back pain by factors such as practice region. This is further complicated by the difficulty of diagnosing and understanding which cases should be referred for imaging or further investigation. Future work can be dedicated to understanding why these variations or inequalities exist and what can be done to standardise management for back pain, and to understanding how clinical tools can aid in the assessment of a suitable candidate for referral to maximise the efficient use of resources; this is an ongoing work implemented in various settings [62, 63, 65–67].

## Conclusions

In our study, we found that more than half of older adults who present to GP surgery practices for an index consultation of back pain were prescribed pain medications, with few referrals to radiology, physiotherapy and other non-pharmacological treatments, clinics or services. Approximately one in every three patients return for repeated consultations, and less than one in five of these patients receive referrals to a new non-pharmacological management modality. Weak opioids with paracetamol/ibuprofen and non-topical NSAIDs are the most commonly prescribed pain medications with more than 70% of prescriptions and patients associated with incident use. There are large variations in non-pharmacological management by practice region, age groups and socioeconomic status, which should be further examined in future studies.

## Supplementary Information

Supplementary Material 1.

## Data Availability

The data that support the findings of this study are available from Clinical Practice Research Datalink, but restrictions apply to the availability of these data, which were used under license for the current study, and so are not publicly available. Codes and code lists are available on GitHub: https://github.com/aaronjyyap/back-pain-aurum.

## Abbreviations

BMI: Body mass index
CCI: Charlson comorbidity index
COVID-19: Coronavirus disease 2019
CPRD: Clinical Practice Research Datalink
EPaMsk: Exercise, physical activity and musculoskeletal
FCP: First contact practitioners
ID: Identification
IMD: Index of multiple deprivation
GP: General practice
NICE: National Institute for Health and Care Excellence
NSAIDs: Non-steroidal anti-inflammatory drugs
RCT: Randomised clinical trial
SES: Socioeconomic status
SMD: Standardised mean difference
