# Supplementary material for: Management of older adults consulting in GP surgery practices with back pain in UK Clinical Practice Research Datalink Aurum: population based study
Source: BMC Musculoskelet Disord. 2026 Mar 2;27:261. doi: 10.1186/s12891-026-09639-7 (PMC13032562; doi:10.1186/s12891-026-09639-7)
Supplement: Supplementary file 1 — Supplementary Material 1. [file 12891_2026_9639_MOESM1_ESM.docx]

**Management of older adults consulting in GP surgery practices with back pain in UK Clinical Practice Research Datalink Aurum: population based study**

*Supplementary Material*

[**Supplementary Table 1**. Back pain diagnoses codes 2](#_Toc219823705)

[**Supplementary Table 2**. List of covariates 3](#_Toc219823706)

[**Supplementary Text 1**. Covariates ascertainment 4](#_Toc219823707)

[**Supplementary Table 3**. Outcomes ascertainment 5](#_Toc219823708)

[**Supplementary Table 4**. Pain medication prescriptions inclusion and exclusion criteria 6](#_Toc219823709)

[**Supplementary Table 5**. Time to the first prescription for pain for each medication class during 18-months follow-up 6](#_Toc219823710)

[**Supplementary Table 6**. Time to the first referral to radiology during 18-months follow-up, stratified by age, gender, SES and comorbidity burden 7](#_Toc219823711)

[**Supplementary Table 7**. Time to the first referral to physiotherapy during 18-months follow-up, stratified by age, gender, SES and comorbidity burden 7](#_Toc219823712)

[**Supplementary Table 8**. Time to the first referral to exercise or physical activity programme, musculoskeletal clinics and services during 18-months follow-up, stratified by age, gender, SES and comorbidity burden 8](#_Toc219823713)

[**Supplementary Table 9**. Time to the first referral to other clinics and services for further assessment during 18-months follow-up, stratified by age, gender, SES and comorbidity burden 8](#_Toc219823714)

[**Supplementary Table 10**. Time to the first prescription for pain medications during 18-months follow-up 9](#_Toc219823715)

[**Supplementary Table 11**. Cox regression models of covariates and time to first referrals and pain medication prescriptions 10](#_Toc219823716)

[**Supplementary Table 12**. Stratified analyses for time to the first referral to exercise or physical activity programme and musculoskeletal clinics and services during 18-months follow-up 12](#_Toc219823717)

[**Supplementary Table 13**. Stratified cox regression models for exercise or physical activity programmes and musculoskeletal clinics and services 13](#_Toc219823718)

# **Supplementary Table 1**. Back pain diagnoses codes

| **Medical Code ID in CPRD Aurum** | **SNOMED-CT Concept ID** | **Term** |
| --- | --- | --- |
| 252314011 | 161894002 | C/O - low back pain |
| 415888015 | 278860009 | Chronic low back pain |
| 416139010 | 279039007 | Low back pain |
| 416146018 | 279040009 | Mechanical low back pain |
| 455771000006114 | 278862001 | Acute low back pain |
| 1573941000006111 | 279038004 | Thoracic back pain |
| 501991000006110 | 161891005 | Back pain |
| 455791000006110 | 279035001 | Acute thoracic back pain |
| 400283013 | 267981009 | Pain in thoracic spine |
| 400284019 | 267982002 | Pain in lumbar spine |
| 216625013 | 135860001 | Exacerbation of backache |
| 252311015 | 161891005 | Backache |
| 320308011 | 209565008 | Lumbar back sprain |
| 320318018 | 281598004 | Back sprain |
| 409930017 | 274162005 | Thoracic back sprain |
| 106921000006111 | 274162005 | Thoracic back sprain |
| 252315012 | 161891005 | Back pain without radiation NOS |
| 2197471000000111 | 847561000000101 | Low back pain clinical pathway |
| 415893017 | 278862001 | Acute back pain - lumbar |
| 369379014 | 247366003 | Acute back pain with sciatica |
| 455801000006111 | 161891005 | Acute back pain - unspecified |
| 252313017 | 161893008 | Back pain worse on sneezing |
| 252312010 | 161892003 | Backache with radiation |
| 252317016 | 161891005 | Backache symptom NOS |
| 311225015 | 302258001 | Back problem |
| 372799017 | 249921008 | Back stiffness |
| 400286017 | 161891005 | Backache, unspecified |
| 252316013 | 161896000 | C/O - upper back ache |
| 311252013 | 202794004 | Lumbago with sciatica |
| 320307018 | 209565008 | Lumbar sprain |
| 416142016 | 279039007 | Lumbalgia |
| 416143014 | 279039007 | Lumbago |
| 502061000006113 | 161891005 | Backache symptom |
| 890991000006119 | 643261000000101 | Backache NOS |
| 896451000006116 | 684561000000107 | Sprain - back |
| 4545111000006119 | 161891005 | Back ache |

# **Supplementary Table 2**. List of covariates

| **Covariates** | **Type** | **Categories** |
| --- | --- | --- |
| Age at index consultation | Numeric | - |
| Age group at index consultation | Categorical | 50-59 years, 60-69 years, 70-79 years, 80 years and older |
| Gender | Categorical | Male, Female, Unknown |
| Ethnicity | Categorical | White, South Asian, Black, Other, Mixed, Unknown |
| Body mass index (BMI) | Categorical | Underweight (BMI<18.5), Healthy range (18.5≤BMI<25), Overweight (25≤BMI<30), Obesity (30≤BMI<40), Severe obesity (BMI≥40) |
| Socioeconomic status  (Practice level Index of Multiple Deprivation as a proxy) | Categorical | Quintile 1 (IMD decile 1-2), Quintile 2 (IMD decile 3-4), Quintile 3 (IMD decile 5-6), Quintile 4 (IMD decile 7-8), Quintile 5 (IMD decile 9-10) |
| Smoking status | Categorical | Non-smoker, Ex-smoker, Active smoker, Unknown |
| Alcohol use | Categorical | Non-drinker, Low, Moderate, High, Unknown |
| Comorbidity burden (CCI as a proxy) | Categorical | None (CCI score of 0), Low (CCI score of 1-2), High (CCI score ≥3) |
| Osteoporosis | Categorical | Yes, No |
| Vertebral fracture | Categorical | Yes, No |
| Practice region | Categorical | North East, North West, Yorkshire And The Humber, East Midlands, West Midlands, East of England, London, South East, South West, Northern Ireland |

# **Supplementary Text 1**. Covariates ascertainment

Body mass index (BMI) was calculated by $BMI=\frac{Weight}{{Height}^{2}}$. All weight records <20kg and height records outside the range of 121-214cm were dropped. Weight records with height recorded on the same date were directly converted to BMI. Remaining weight records with an older height record available were converted to BMI using the most recent height data, followed by weight records with a future height record available. If no height and/or weight records were available but a BMI value was available in the database, the recorded BMI value was used. Finally, BMI values outside the range of 9-180kg/m^2^ were dropped. The most recent BMI value from the index consultation date was retained as the final value used in the analysis.

For smoking status, the nearest coded record at any time before (from 1995) or equal to the index consultation date was retained for each patient. If there were different categories on the same date, we took the next most recent record of smoking status, otherwise patient was coded as having unknown smoking status.

For alcohol use, the nearest coded record at any time before (from 1995) or equal to the index consultation date was retained. If there were different categories coded on the same date, we retained the highest level of alcohol use. Alcohol use was used to describe the cohort but was not added to the Cox regression models due to high missingness in the data.

# **Supplementary Table 3**. Outcomes ascertainment

| **SN** | **Outcome** | **Description** |
| --- | --- | --- |
| 1 | Referrals to radiology | Any record of a referral for a radiology procedure made during the same consultation visit or on the same day of a consultation for back pain |
| 2 | Referrals to physiotherapy | Any record of a referral to physiotherapy made during the same consultation visit or on the same day of a consultation for back pain |
| 3 | Medication prescription for pain | Any record of a medication used to treat pain prescribed on the same day of a consultation for back pain (paracetamol, non-steroidal anti-inflammatory, opioids, gabapentinoids, benzodiazepines, non-benzodiazepine hypnotics, antidepressants commonly prescribed for pain) |
| 4 | Referrals to external clinics or services for further assessment | Any record of a referral to back pain clinic, falls service or assessment, fracture clinic, general medicine clinic, geriatric or older people services, orthopaedics, osteoporosis clinics, pain clinics, rheumatology or spinal team made during the same consultation visit or on the same day of a consultation for back pain |
| 5 | Referrals to external clinics or services for therapy | Any record of a referral to exercise or physical activity programme, musculoskeletal clinic or services made during the same consultation visit or on the same day of a consultation for back pain |

# **Supplementary Table 4**. Pain medication prescriptions inclusion and exclusion criteria

| **Medication class** | **Inclusion** | **Exclusion** |
| --- | --- | --- |
| Paracetamol | Oral or rectal formulations only | Combination products with drug substances other than paracetamol |
| NSAIDs | Oral or rectal formulations only | Injections, topicals, eye drops, aspirin. Combination products with other drug substances other than NSAIDs |
| Topical NSAIDs | Topical formulations only |  |
| Opioids in combination with paracetamol or ibuprofen | Codeine, tramadol, dextropropoxyphene in combination with paracetamol or codeine in combination with ibuprofen. Oral formulations only |  |
| Opioids | Any oral, buccal, sublingual, subcutaneous, transdermal formulations containing opioids | Codeine, tramadol, dextropropoxyphene in combination with paracetamol or codeine in combination with ibuprofen |
| Gabapentinoids | Oral formulations of gabapentin or pregabalin |  |
| Antidepressants (for pain) | Oral formulations of amitriptyline, duloxetine, fluoxetine, paroxetine, citalopram, sertraline, nortriptyline |  |
| Benzodiazepines | Oral formulations only. | Rectal formulations (seizures), injection formulations (psychosis) |
| Non-benzodiazepine hypnotics | Oral formulations of zopiclone, zolpidem, clomethiazole |  |

# **Supplementary Table 5**. Time to the first prescription for pain for each medication class during 18-months follow-up

|  | **Patients with pain medication prescriptions, N (%)** | **Follow-up time (100 py)** | **Pr of pain Rx at 0-days, %** | **Pr of pain Rx at 180-days, %** | **Pr of pain Rx at 365-days, %** |
| --- | --- | --- | --- | --- | --- |
| Paracetamol only | 51533 (8.7) | 7873 | 5.9 | 7.7 | 8.3 |
| NSAIDs only | 127164 (21.4) | 6790 | 16.8 | 19.7 | 20.7 |
| Topical NSAIDs | 67031 (11.3) | 7648 | 8.5 | 10.1 | 10.8 |
| Opioids in combination with paracetamol or ibuprofen | 151390 (25.5) | 6493 | 20.0 | 23.4 | 24.6 |
| Opioids only | 101713 (17.1) | 7223 | 11.8 | 15.3 | 16.4 |
| Gabapentinoids | 35908 (6.0) | 8092 | 3.0 | 4.9 | 5.6 |
| Antidepressants (for pain) | 60786 (10.2) | 7758 | 6.1 | 8.7 | 9.6 |
| Benzodiazepines | 49655 (8.4) | 7864 | 6.2 | 7.5 | 8.0 |
| Non-benzodiazepines hypnotics | 6281 (1.1) | 8448 | 0.7 | 0.9 | 1.0 |

Pr: Probability; py: person-years

# **Supplementary Table 6**. Time to the first referral to radiology during 18-months follow-up, stratified by age, gender, SES and comorbidity burden

|  | **Total N** | **Patient referrals, N (%)** | **Follow-up time (100 py)** | **Pr of referral at 0-days, %** | **Pr of referral at 180-days, %** | **Pr of referral at 365-days, %** |
| --- | --- | --- | --- | --- | --- | --- |
| **Time to first referral to radiology** | | | | | | |
| Overall | 594559 | 23712 (4.0) | 8218 | 2.6 | 3.5 | 3.8 |
| Age groups, years  50-59  60-69  70-79  ≥ 80 | 199982  173931  136448  84198 | 5766 (2.9)  6805 (3.9)  6694 (4.9)  4447 (5.3) | 2837  2436  1870  1075 | 1.9  2.6  3.2  3.4 | 2.5  3.5  4.4  4.8 | 2.7  3.7  4.7  5.1 |
| Gender  Male  Female | 255253  339306 | 8749 (3.4)  14963 (4.4) | 3538  4680 | 2.2  2.9 | 3.1  3.9 | 3.4  4.2 |
| Socioeconomic status  Quintile 1 (Lowest)  Quintile 2  Quintile 3  Quintile 4  Quintile 5 (Highest) | 100981  96920  117672  136794  142192 | 3873 (3.8)  4093 (4.2)  4845 (4.1)  6039 (4.4)  4862 (3.4) | 1402  1342  1623  1883  1968 | 2.5  2.7  2.7  2.8  2.2 | 3.4  3.7  3.7  3.9  3.0 | 3.7  4.0  3.9  4.2  3.2 |
| Comorbidity burden  High (CCI score ≥ 3)  Low (CCI score 1-2)  None (CCI score 0) | 16994  162479  415086 | 831 (4.9)  7184 (4.4)  15697 (3.8) | 208  2194  5816 | 3.0  2.9  2.5 | 4.3  3.9  3.3 | 4.7  4.2  3.6 |

Pr: Probability; py: person-years

# **Supplementary Table 7**. Time to the first referral to physiotherapy during 18-months follow-up, stratified by age, gender, SES and comorbidity burden

|  | **Total N** | **Patient referrals, N (%)** | **Follow-up time (100 py)** | **Pr of referral at 0-days, %** | **Pr of referral at 180-days, %** | **Pr of referral at 365-days, %** |
| --- | --- | --- | --- | --- | --- | --- |
| **Time to first referral to physiotherapy** | | | | | | |
| Overall | 594559 | 2856 (0.5) | 8489 | 0.3 | 0.4 | 0.5 |
| Age groups, years  50-59  60-69  70-79  ≥ 80 | 199982  173931  136448  84198 | 1081 (0.5)  890 (0.5)  581 (0.4)  304 (0.4) | 2898  2514  1950  1127 | 0.4  0.3  0.3  0.2 | 0.5  0.4  0.4  0.3 | 0.5  0.5  0.4  0.4 |
| Gender  Male  Female | 255253  339306 | 1204 (0.5)  1652 (0.5) | 3636  4853 | 0.3  0.3 | 0.4  0.4 | 0.4  0.5 |
| Socioeconomic status  Quintile 1 (Lowest)  Quintile 2  Quintile 3  Quintile 4  Quintile 5 (Highest) | 100981  96920  117672  136794  142192 | 441 (0.4)  346 (0.4)  490 (0.4)  755 (0.6)  824 (0.6) | 1447  1391  1680  1952  2020 | 0.3  0.2  0.3  0.3  0.4 | 0.4  0.3  0.4  0.5  0.5 | 0.4  0.3  0.4  0.5  0.5 |
| Comorbidity burden  High (CCI score ≥ 3)  Low (CCI score 1-2)  None (CCI score 0) | 16994  162479  415086 | 51 (0.3)  721 (0.4)  2084 (0.5) | 217  2278  5995 | 0.2  0.3  0.3 | 0.3  0.4  0.4 | 0.3  0.4  0.5 |

Pr: Probability; py: person-years

# **Supplementary Table 8**. Time to the first referral to exercise or physical activity programme, musculoskeletal clinics and services during 18-months follow-up, stratified by age, gender, SES and comorbidity burden

|  | **Total N** | **Patient referrals, N (%)** | **Follow-up time (100 py)** | **Pr of referral at 0-days, %** | **Pr of referral at 180-days, %** | **Pr of referral at 365-days, %** |
| --- | --- | --- | --- | --- | --- | --- |
| **Time to first referral to exercise or physical activity programme, musculoskeletal clinics and services** | | | | | | |
| Overall | 594559 | 22182 (3.7) | 8251 | 1.7 | 3.1 | 3.4 |
| Age groups, years  50-59  60-69  70-79  ≥ 80 | 199982  173931  136448  84198 | 8629 (4.3)  6980 (4.0)  4771 (3.5)  1802 (2.1) | 2805  2439  1899  1109 | 2.0  1.8  1.5  0.9 | 3.5  3.3  2.9  1.8 | 4.0  3.7  3.2  2.0 |
| Gender  Male  Female | 255253  339306 | 9239 (3.6)  12943 (3.8) | 3537  4714 | 1.6  1.7 | 3.0  3.1 | 3.3  3.5 |
| Socioeconomic status  Quintile 1 (Lowest)  Quintile 2  Quintile 3  Quintile 4  Quintile 5 (Highest) | 100981  96920  117672  136794  142192 | 4201 (4.2)  3440 (3.5)  4380 (3.7)  5057 (3.7)  5104 (3.6) | 1400  1352  1632  1899  1968 | 1.9  1.7  1.7  1.6  1.5 | 3.4  3.0  3.1  3.0  2.9 | 3.8  3.3  3.4  3.4  3.3 |
| Comorbidity burden  High (CCI score ≥ 3)  Low (CCI score 1-2)  None (CCI score 0) | 16994  162479  415086 | 470 (2.8)  5561 (3.4)  16151 (3.9) | 212  2219  5820 | 1.1  1.5  1.8 | 2.3  2.8  3.2 | 2.6  3.1  3.6 |

Pr: Probability; py: person-years

# **Supplementary Table 9**. Time to the first referral to other clinics and services for further assessment during 18-months follow-up, stratified by age, gender, SES and comorbidity burden

|  | **Total N** | **Patient referrals, N (%)** | **Follow-up time (100 py)** | **Pr of referral at 0-days, %** | **Pr of referral at 180-days, %** | **Pr of referral at 365-days, %** |
| --- | --- | --- | --- | --- | --- | --- |
| **Time to first referral to other clinics and services for further assessment** | | | | | | |
| Overall | 594559 | 20755 (3.5) | 8272 | 1.3 | 2.8 | 3.2 |
| Age groups, years  50-59  60-69  70-79  ≥ 80 | 199982  173931  136448  84198 | 7522 (3.8)  6367 (3.7)  4778 (3.5)  2088 (2.5) | 2820  2447  1899  1106 | 1.4  1.4  1.3  0.9 | 3.0  3.0  2.9  2.1 | 3.5  3.4  3.4  2.3 |
| Gender  Male  Female | 255253  339306 | 8981 (3.5)  11774 (3.5) | 3541  4731 | 1.4  1.3 | 2.9  2.8 | 3.4  3.4 |
| Socioeconomic status  Quintile 1 (Lowest)  Quintile 2  Quintile 3  Quintile 4  Quintile 5 (Highest) | 100981  96920  117672  136794  142192 | 4283 (4.2)  3824 (3.9)  3891 (3.3)  4663 (3.4)  4094 (2.9) | 1400  1348  1639  1905  1981 | 1.6  1.6  1.3  1.2  1.1 | 3.5  3.4  2.7  2.8  2.3 | 3.9  3.7  3.1  3.2  2.7 |
| Comorbidity burden  High (CCI score ≥ 3)  Low (CCI score 1-2)  None (CCI score 0) | 16994  162479  415086 | 491 (2.9)  5198 (3.2)  15066 (3.6) | 212  2224  5836 | 0.9  1.2  1.4 | 2.4  2.6  3.0 | 2.8  3.0  3.4 |

Pr: Probability; py: person-years

# **Supplementary Table 10**. Time to the first prescription for pain medications during 18-months follow-up

|  | **Total N** | **Patients with pain medication prescriptions, N (%)** | **Follow-up time (100 py)** | **Pr of pain Rx at 0-days, %** | **Pr of pain Rx at 180-days, %** | **Pr of pain Rx at 365-days, %** |
| --- | --- | --- | --- | --- | --- | --- |
| **Time to the first prescription for pain** | | | | | | |
| Overall | 594559 | 381829 (64.2) | 3249 | 56.4 | 61.5 | 63.2 |
| Age groups, years  50-59  60-69  70-79  ≥ 80 | 199982  173931  136448  84198 | 126545 (63.3)  111395 (64.0)  87734 (64.3)  56155 (66.7) | 1135  964  748  403 | 55.8  56.6  56.0  58.0 | 60.6  61.4  61.4  64.2 | 62.2  63.0  63.2  65.8 |
| Gender  Male  Female | 255253  339306 | 161569 (63.3)  220260 (64.9) | 1422  1827 | 55.9  56.8 | 60.8  62.1 | 62.3  63.8 |
| Socioeconomic status  Quintile 1 (Lowest)  Quintile 2  Quintile 3  Quintile 4  Quintile 5 (Highest) | 100981  96920  117672  136794  142192 | 60424 (59.8)  60410 (62.3)  74328 (63.2)  90153 (65.9)  96514 (67.9) | 618  559  661  714  698 | 52.2  54.7  55.3  57.9  60.0 | 57.2  59.6  60.5  61.2  65.1 | 58.8  61.3  62.1  64.8  66.8 |
| Comorbidity burden  High (CCI score ≥ 3)  Low (CCI score 1-2)  None (CCI score 0) | 16994  162479  415086 | 11336 (66.7)  110035 (67.7)  260458 (62.7) | 78  794  2377 | 57.5  59.2  55.2 | 64.0  64.8  60.1 | 66.0  66.7  61.7 |

Pr: Probability; py: person-years

# **Supplementary Table 11**. Cox regression models of covariates and time to first referrals and pain medication prescriptions

|  | **Radiology** | | **Physiotherapy** | | **Exercise, physical activity programmes and**  **musculoskeletal clinics services** | | **Other clinics and services for further assessment** | | **Pain medication prescriptions** | |
| --- | --- | --- | --- | --- | --- | --- | --- | --- | --- | --- |
|  | **n** | **Adj HR (95% CI)** | **n** | **Adj HR (95% CI)** | **n** | **Adj HR (95% CI)** | **n** | **Adj HR (95% CI)** | **n** | **Adj HR (95% CI)** |
| **Age** |  |  |  |  |  |  |  |  |  |  |
| 50-59 years | 5766 | 1 | 1081 | 1 | 8629 | 1 | 7522 | 1 | 126545 | 1 |
| 60-69 years | 6805 | 1.39 (1.34-1.44) | 890 | 0.98 (0.89-1.07) | 6980 | 0.95 (0.92-0.98) | 6367 | 0.97 (0.94-1.00) | 111395 | 1.03 (1.02-1.04) |
| 70-79 years | 6694 | 1.74 (1.68-1.81) | 581 | 0.84 (0.76-0.94) | 4771 | 0.84 (0.81-0.87) | 4778 | 0.93 (0.89-0.96) | 87734 | 1.04 (1.04-1.05) |
| 80 years and older | 4447 | 1.87 (1.79-1.95) | 304 | 0.74 (0.65-0.85) | 1802 | 0.53 (0.50-0.56) | 2088 | 0.67 (0.64-0.70) | 56155 | 1.14 (1.13-1.16) |
| **Gender** |  |  |  |  |  |  |  |  |  |  |
| Male | 8749 | 1 | 1204 | 1 | 9239 | 1 | 8981 | 1 | 161569 | 1 |
| Female | 14963 | 1.26 (1.23-1.29) | 1652 | 1.04 (0.97-1.13) | 12943 | 1.08 (1.05-1.11) | 11774 | 0.99 (0.96-1.02) | 220260 | 1.05 (1.05-1.06) |
| **Ethnicity** |  |  |  |  |  |  |  |  |  |  |
| White | 20469 | 1 | 2435 | 1 | 19174 | 1 | 18540 | 1 | 335667 | 1 |
| South Asian | 1552 | 0.92 (0.87-0.97) | 221 | 1.27 (1.09-1.47) | 1203 | 0.84 (0.79-0.89) | 1210 | 0.96 (0.90-1.02) | 24275 | 1.16 (1.14-1.17) |
| Black | 1189 | 1.14 (1.07-1.22) | 132 | 1.18 (0.98-1.43) | 1219 | 1.22 (1.15-1.30) | 639 | 0.82 (0.75-0.89) | 15051 | 1.10 (1.08-1.12) |
| Other | 320 | 1.11 (0.99-1.24) | 36 | 1.13 (0.81-1.58) | 367 | 1.27 (1.14-1.41) | 214 | 0.89 (0.78-1.02) | 4102 | 1.01 (0.98-1.04) |
| Mixed | 182 | 0.97 (0.84-1.13) | 32 | 1.49 (1.05-2.12) | 219 | 1.17 (1.02-1.33) | 152 | 0.95 (0.81-1.11) | 2734 | 0.99 (0.95-1.03) |
| **BMI** |  |  |  |  |  |  |  |  |  |  |
| Underweight (BMI<18.5) | 430 | 1.10 (1.00-1.22) | 31 | 0.85 (0.59-1.22) | 208 | 0.79 (0.69-0.91) | 193 | 0.76 (0.66-0.87) | 5397 | 1.05 (1.02-1.08) |
| Healthy range (18.5≤BMI<25) | 7045 | 1 | 790 | 1 | 5715 | 1 | 5540 | 1 | 100411 | 1 |
| Overweight (25≤BMI<30) | 8628 | 0.93 (0.90-0.96) | 1073 | 0.98 (0.89-1.07) | 8266 | 1.06 (1.03-1.10) | 7813 | 1.04 (1.00-1.07) | 141617 | 1.07 (1.06-1.08) |
| Obesity (30≤BMI<40) | 6728 | 0.92 (0.89-0.95) | 842 | 0.96 (0.87-1.06) | 6974 | 1.12 (1.08-1.16) | 6336 | 1.08 (1.04-1.12) | 117031 | 1.15 (1.15-1.16) |
| Severe obesity | 881 | 0.87 (0.81-0.94) | 120 | 0.93 (0.76-1.12) | 1019 | 1.11 (1.04-1.19) | 873 | 1.04 (0.97-1.12) | 17373 | 1.24 (1.22-1.26) |
| **SES** |  |  |  |  |  |  |  |  |  |  |
| Quintile 1 (Lowest) | 3873 | 1 | 441 | 1 | 4201 | 1 | 4283 | 1 | 60424 | 1 |
| Quintile 2 | 4093 | 1.09 (1.04-1.14) | 346 | 0.90 (0.78-1.04) | 3440 | 0.84 (0.80-0.88) | 3824 | 0.98 (0.94-1.03) | 60410 | 1.05 (1.04-1.06) |
| Quintile 3 | 4845 | 1.02 (0.97-1.06) | 490 | 1.06 (0.93-1.20) | 4380 | 0.86 (0.82-0.90) | 3891 | 0.82 (0.79-0.86) | 74328 | 1.06 (1.05-1.07) |
| Quintile 4 | 6039 | 1.09 (1.04-1.13) | 755 | 1.47 (1.30-1.67) | 5057 | 0.83 (0.79-0.86) | 4663 | 0.89 (0.85-0.93) | 90153 | 1.12 (1.10-1.13) |
| Quintile 5 (Highest) | 4862 | 0.90 (0.86-0.94) | 824 | 1.66 (1.46-1.88) | 5104 | 0.80 (0.77-0.84) | 4094 | 0.79 (0.75-0.83) | 96514 | 1.15 (1.14-1.17) |
| **Region** |  |  |  |  |  |  |  |  |  |  |
| North East | 122 | 0.13 (0.11-0.16) | 155 | 1.75 (1.46-2.11) | 1448 | 1.37 (1.29-1.45) | 730 | 0.89 (0.82-0.96) | 17494 | 0.97 (0.96-0.99) |
| North West | 4299 | 1 | 427 | 1 | 5070 | 1 | 3889 | 1 | 84164 | 1 |
| Yorkshire And The Humber | 670 | 0.94 (0.87-1.02) | 186 | 2.78 (2.34-3.31) | 992 | 1.17 (1.09-1.25) | 725 | 1.11 (1.03-1.20) | 13904 | 1.02 (1.00-1.04) |
| East Midlands | 233 | 0.46 (0.41-0.53) | 127 | 2.99 (2.45-3.65) | 91 | 0.15 (0.12-0.18) | 459 | 0.97 (0.88-1.07) | 9399 | 1.01 (0.99-1.03) |
| West Midlands | 6058 | 1.77 (1.70-1.84) | 504 | 1.48 (1.30-1.68) | 2370 | 0.57 (0.55-0.60) | 3306 | 1.05 (1.00-1.10) | 69012 | 1.04 (1.03-1.05) |
| East of England | 909 | 1.00 (0.93-1.07) | 156 | 2.25 (1.86-2.72) | 877 | 0.77 (0.72-0.83) | 1236 | 1.43 (1.34-1.53) | 16164 | 0.98 (0.96-1.00) |
| London | 5431 | 1.80 (1.72-1.88) | 438 | 1.40 (1.21-1.62) | 4796 | 1.26 (1.21-1.32) | 3155 | 1.14 (1.08-1.20) | 59869 | 0.99 (0.98-1.00) |
| South East | 5077 | 1.26 (1.21-1.32) | 617 | 1.97 (1.73-2.25) | 4765 | 0.97 (0.93-1.01) | 5297 | 1.40 (1.34-1.46) | 72409 | 0.97 (0.96-0.98) |
| South West | 840 | 0.38 (0.36-0.41) | 245 | 1.34 (1.14-1.57) | 1763 | 0.70 (0.66-0.74) | 1913 | 0.98 (0.93-1.04) | 38611 | 0.93 (0.92-0.94) |
| Northern Ireland | 73 | 2.09 (1.65-2.63) | <5 | 0.25 (0.04-1.79) | 10 | 0.22 (0.12-0.41) | 45 | 1.35 (1.01-1.81) | 803 | 1.20 (1.12-1.29) |
| **Smoking status** |  |  |  |  |  |  |  |  |  |  |
| Non-smoker | 12669 | 1 | 1525 | 1 | 11504 | 1 | 11011 | 1 | 193249 | 1 |
| Ex-smoker | 7557 | 1.02 (0.99-1.05) | 880 | 0.99 (0.91-1.08) | 7013 | 1.04 (1.01-1.07) | 6596 | 0.97 (0.94-1.01) | 123876 | 1.07 (1.06-1.07) |
| Active smoker | 3486 | 1.09 (1.05-1.14) | 451 | 0.96 (0.86-1.07) | 3665 | 1.05 (1.01-1.09) | 3148 | 0.97 (0.93-1.01) | 64704 | 1.28 (1.26-1.29) |
| **Comorbidity burden** |  |  |  |  |  |  |  |  |  |  |
| None (CCI score 0) | 15697 | 1 | 2084 | 1 | 16151 | 1 | 15066 | 1 | 260458 | 1 |
| Low (CCI score 1-2) | 7184 | 1.07 (1.04-1.11) | 721 | 0.93 (0.85-1.01) | 5561 | 0.96 (0.93-0.99) | 5198 | 0.95 (0.92-0.98) | 110035 | 1.07 (1.06-1.08) |
| High (CCI score ≥ 3) | 831 | 1.19 (1.11-1.28) | 51 | 0.69 (0.52-0.91) | 470 | 0.87 (0.79-0.96) | 491 | 0.95 (0.86-1.04) | 11336 | 1.04 (1.02-1.06) |
| **Osteoporosis** |  |  |  |  |  |  |  |  |  |  |
| No | 23122 | 1 | 2824 | 1 | 21862 | 1 | 20386 | 1 | 375595 | 1 |
| Yes | 590 | 1.25 (1.15-1.35) | 32 | 0.78 (0.55-1.10) | 320 | 1.04 (0.93-1.17) | 369 | 1.23 (1.11-1.36) | 6234 | 0.99 (0.96-1.01) |
| **Vertebral fracture** |  |  |  |  |  |  |  |  |  |  |
| No | 23691 | 1 | 2855 | 1 | 22175 | 1 | 20738 | 1 | 381532 | 1 |
| Yes | 21 | 0.91 (0.59-1.40) | <5 | 0.61 (0.09-4.31) | 7 | 0.57 (0.27-1.19) | 17 | 1.26 (0.78-2.04) | 297 | 1.04 (0.93-1.17) |

Adj HR: Adjusted hazard ratio; BMI: body mass index; CCI: Charlson comorbidity index; SES: socioeconomic status.

# **Supplementary Table 12**. Stratified analyses for time to the first referral to exercise and physical activity programme and musculoskeletal clinics and services during 18-months follow-up

|  | **Total N** | **Patient referrals, N (%)** | **Follow-up time (100 py)** | **Pr of referral at 0-days, %** | **Pr of referral at 180-days, %** | **Pr of referral at 365-days, %** |
| --- | --- | --- | --- | --- | --- | --- |
| Combined | 594559 | 22182 (3.7) | 8251 | 1.7 | 3.1 | 3.4 |
| Exercise and physical activity programmes | 594559 | 498 (<0.1) | 8520 | <0.1 | <0.1 | <0.1 |
| Musculoskeletal clinics and services | 594559 | 21736 (3.7) | 8257 | 1.6 | 3.0 | 3.4 |

Pr: Probability; py: person-years

# **Supplementary Table 13**. Stratified cox regression models for exercise and physical activity programmes and musculoskeletal clinics and services

|  | **Exercise and physical activity programmes** | | **Musculoskeletal clinics and services** | |
| --- | --- | --- | --- | --- |
|  | **n** | **Adj HR (95% CI)** | **n** | **Adj HR (95% CI)** |
| **Age** |  |  |  |  |
| 50-59 years | 235 | 1 | 8415 | 1 |
| 60-69 years | 160 | 0.84 (0.69-1.03) | 6837 | 0.96 (0.93-0.99) |
| 70-79 years | 75 | 0.54 (0.41-0.71) | 4708 | 0.85 (0.82-0.88) |
| 80 years and older | 28 | 0.39 (0.26-0.59) | 1776 | 0.53 (0.51-0.56) |
| **Gender** |  |  |  |  |
| Male | 173 | 1 | 9086 | 1 |
| Female | 325 | 1.41 (1.17-1.70) | 12650 | 1.07 (1.04-1.10) |
| **Ethnicity** |  |  |  |  |
| White | 352 | 1 | 18861 | 1 |
| South Asian | 52 | 2.02 (1.46-2.78) | 1156 | 0.82 (0.77-0.87) |
| Black | 74 | 3.09 (2.28-4.19) | 1151 | 1.18 (1.11-1.26) |
| Other | 12 | 2.25 (1.25-4.05) | 357 | 1.25 (1.13-1.39) |
| Mixed | 8 | 2.04 (1.00-4.14) | 211 | 1.15 (1.00-1.31) |
| **BMI** |  |  |  |  |
| Underweight (BMI<18.5) | <5 | 0.31 (0.04-2.24) | 207 | 0.80 (0.69-0.91) |
| Healthy range (18.5≤BMI<25) | 73 | 1 | 5653 | 1 |
| Overweight (25≤BMI<30) | 133 | 1.33 (1.00-1.77) | 8148 | 1.06 (1.02-1.10) |
| Obesity (30≤BMI<40) | 225 | 2.66 (2.04-3.48) | 6768 | 1.10 (1.06-1.14) |
| Severe obesity | 66 | 4.98 (3.54-7.01) | 960 | 1.06 (0.99-1.13) |
| **SES** |  |  |  |  |
| Quintile 1 (Lowest) | 76 | 1 | 4133 | 1 |
| Quintile 2 | 64 | 0.88 (0.63-1.24) | 3380 | 0.83 (0.80-0.87) |
| Quintile 3 | 107 | 1.10 (0.81-1.49) | 4282 | 0.85 (0.82-0.89) |
| Quintile 4 | 128 | 0.94 (0.69-1.27) | 4942 | 0.83 (0.79-0.86) |
| Quintile 5 (Highest) | 123 | 0.88 (0.64-1.21) | 4999 | 0.80 (0.77-0.84) |
| **Region** |  |  |  |  |
| North East | 30 | 1.36 (0.90-2.04) | 1421 | 1.36 (1.28-1.44) |
| North West | 106 | 1 | 4987 | 1 |
| Yorkshire And The Humber | 29 | 1.66 (1.10-2.50) | 965 | 1.15 (1.08-1.24) |
| East Midlands | <5 | 0.16 (0.04-0.66) | 89 | 0.15 (0.12-0.18) |
| West Midlands | 25 | 0.27 (0.17-0.41) | 2346 | 0.58 (0.55-0.61) |
| East of England | 35 | 1.64 (1.09-2.45) | 844 | 0.76 (0.70-0.82) |
| London | 167 | 1.37 (1.04-1.81) | 4642 | 1.25 (1.20-1.31) |
| South East | 76 | 0.76 (0.55-1.03) | 4694 | 0.97 (0.93-1.01) |
| South West | 28 | 0.54 (0.36-0.82) | 1738 | 0.70 (0.66-0.74) |
| Northern Ireland | 0 | - | 10 | 0.22 (0.12-0.42) |
| **Smoking status** |  |  |  |  |
| Non-smoker | 271 | 1 | 11257 | 1 |
| Ex-smoker | 153 | 1.19 (0.96-1.46) | 6879 | 1.03 (1.00-1.07) |
| Active smoker | 74 | 1.09 (0.83-1.42) | 3600 | 1.05 (1.01-1.09) |
| **Comorbidity burden** |  |  |  |  |
| None (CCI score 0) | 338 | 1 | 15850 | 1 |
| Low (CCI score 1-2) | 147 | 1.09 (0.89-1.34) | 5428 | 0.95 (0.92-0.99) |
| High (CCI score ≥ 3) | 13 | 1.28 (0.73-2.24) | 458 | 0.86 (0.79-0.95) |
| **Osteoporosis** |  |  |  |  |
| No | 495 | 1 | 21419 | 1 |
| Yes | <5 | 0.60 (0.19-1.88) | 317 | 1.05 (0.94-1.17) |
| **Vertebral fracture** |  |  |  |  |
| No | 498 | 1 | 21729 | 1 |
| Yes | 0 | - | 7 | 0.58 (0.27-1.21) |

Adj HR: Adjusted hazard ratio; BMI: body mass index; CCI: Charlson comorbidity index; SES: socioeconomic status.
